# Efficacy of automated fasteners versus hand-tied knots in cardiac surgery: a systematic review and meta-analysis

**DOI:** 10.17179/excli2023-6885

**Published:** 2024-02-06

**Authors:** Zoaib Habib Tharwani, Muhammad Abdul Qadeer, Ali Abdullah, Rubab Ali, Muhammad Ahmed Chaudhary, Shurjeel Uddin Qazi, Sameh M. Said

**Affiliations:** 1 Department of Medicine, Dow University of Health Sciences, Karachi, Pakistan; 2 Department of Medicine, Jinnah Sindh Medical University, Karachi, Pakistan; 3 Division of Pediatric and Adult Congenital Cardiac Surgery, Maria Fareri Children’s Hospital, Westchester Medical Center, Valhalla, New York 10696, USA; 4 New York Medical College, 100 Woods Road, Valhalla, New York 10595, USA

**Keywords:** cardiac valvular surgery, COR-KNOT, automated fasteners, hand-tied knots

## Abstract

Valve surgery is common in cardiac procedures, with fasteners like COR-KNOT® and hand-tied knots used for knot securing. This study compares their efficacy in valve surgery patients. We searched PubMed, SCOPUS, and Cochrane Central until August 2023. Outcomes assessed included aortic cross-clamp time (AXT), cardiopulmonary bypass (CPB) time, valvular regurgitation, mortality, prolonged ventilatory support, atrial fibrillation, postoperative left ventricular ejection fraction (LVEF), and renal failure. Subgroup analysis was performed for minimally invasive and open cardiac surgery. We used a random effects model for analysis. We included eight observational studies and two randomized controlled trials (RCTs) with a total of 1.411 participants. COR-KNOT significantly reduced AXT [MD -15.14, 95 % CI (-18.57, -11.70), P<0.00001] and CPB time [MD -12.38, 95 % CI (-14.99, -9.77), P<0.00001]. Valvular regurgitation [RR 0.40, 95 % CI (0.26, 0.61), P<0.0001] and need for prolonged ventilatory support [RR 0.29, 95 % CI (0.13, 0.65), P=0.003] were significantly lower with COR-KNOT. There were no significant differences in mortality [RR 0.39, 95 % CI (0.09, 1.69), P=0.44], atrial fibrillation [RR 1.03, 95 % CI (0.83, 1.27), P=0.81], LVEF changes [MD 0.05, 95 % CI (−1.37, 1.47), P = 0.95], or renal failure [RR 0.87, 95 % CI (0.16, 4.80), P = 0.87]. COR-KNOT devices reduce operative time and valvular regurgitation without increasing mortality or adverse outcomes. This supports their use in enhancing surgical efficiency and patient outcomes. However, ongoing discussions about suturing techniques, especially in minimally invasive procedures, highlight the need for further research and consensus among practitioners.

See also the graphical abstract(Fig. 1).

## Introduction

The incidence of degenerative valve disease is on the rise as the general population ages, leading to an increase in the need for surgical interventions to repair or replace these valves. The incidence of valvular diseases in the general population is 11.9 %, with mitral regurgitation being the most common, followed by aortic regurgitation (Matiasz and Rigolin, 2018[9]). Surgery remains the mainstay of treatment for symptomatic patients with severe disease, with excellent long-term outcomes (Reddy and Punjabi, 2007[15]).

A fundamental aspect of heart valve surgery is knot-tying. The traditional method of achieving secure knots is hand-tying (Jha et al., 2007[5]). Hand-tying, however, has several potential drawbacks, such as longer aortic cross-clamp time (AXT) and cardiopulmonary bypass (CPB) time, especially when multivalvular procedures are needed. This may increase the risk of postoperative morbidity and mortality (Ler et al., 2021[7]; Sazzad et al., 2021[19]). Additionally, hand-tying, if insecure, may lead to higher rates of postoperative paravalvular leak or prosthetic dehiscence (Lee et al., 2018[6]).

Automated fasteners, such as the COR-KNOT@ by LSI Solutions® are used in heart valve surgery to eliminate the need for manual tying during prosthetic implantation. It consists of an automated fixture with an articulating arm and a target device holder, as well as one or more additional automated fixtures with suturing arms and needle holders (Sazzad et al., 2021[19]). The device can rotate the target device, allowing the suturing arms to perform operations such as forming sutures without the need for human intervention (Lee et al., 2018[6]). The use of automated fasteners has been shown to reduce AXT and CBP time, leading to shorter overall operative time when compared with hand-tyng (Salmasi et al., 2019[18]; Sazzad et al., 2021[19]; Cody et al., 2021[2]).

While the benefits of automated fasteners are clear, it is crucial to be aware of the potential complications associated with them. Potential concerns include thrombi/clot formation with subsequent systemic embolization, coronary ostial obstruction, infective endocarditis, periprosthetic regurgitation and hemolysis (Sadeghian and Savand-Roomi, 2017[17]).

There continues to be a lack of consensus regarding the role of automated suture fasteners, such as the COR-KNOT device, in the current era and if they are in fact a better technique compared to hand-tying, and therefore should be adopted on a larger scale. They appear to be of greater value in minimally invasive valve surgery or when the surgical field is limited (Perin et al., 2019[13]). Given these critical considerations, this study aims to assess the efficacy of using COR-KNOT devices over hand-tied sutures in valvular surgery.

## Methods

We followed the Preferred Reporting Items for Systematic review and Meta-Analyses (PRISMA) guidelines and the Risk of Bias in Systematic reviews and assessment of multiple systematic reviews (AMSTAR) 2 while performing this meta-analysis (Shea et al., 2017[20]; Page et al., 2021[12]).

### Data sources and search strategy

MEDLINE, EMBASE and Cochrane CENTRAL were comprehensively searched from inception through July 2023 by two independent reviewers (MAC and RA). We extracted studies based on abstracts and titles. A full-text appraisal was sought when required. MeSH phrases and keywords were used to find keywords for “COR-KNOT”, “automated fastener”, “automated suture”, “automated suture fastening device”, “automated titanium fasteners” and “COR-KNOT heart valve surgery”.

### Study selection

#### Data extraction and assessment of study quality

We included studies if they were: (1) randomized controlled trials (RCTs) or analysis of RCTs that determined the impact of automated sutures and hand-tied sutures in different interventional arms, (2) reported either of aortic cross-clamp time (AXT), cardiopulmonary bypass (CBP) time, valvular regurgitation, mortality, prolonged ventilatory support, atrial fibrillation, postoperative left ventricular ejection fraction (LVEF), and renal failure as one of their outcomes, (3) included patients with valvular disease(s) undergoing surgical replacement or repair. A third investigator (AA) was consulted in case of any disagreement regarding study selection. All articles were then uploaded to Endnote Reference Library (Version X7.5; Clarivate Analytics, Philadelphia, Pennsylvania) software to remove any duplicates.

Two reviewers (ZHT and MAQ) independently extracted from the selected studies the characteristics of the studies, patient demographics, summary events, number of events, sample sizes and treatment type. Summary events were also extracted for outcomes of interest, and mean difference (MD) with standard deviation (SD) from baseline. 

The quality assessment of included studies was conducted through Joanna Briggs' Institute (JBI) critical appraisal checklist (Moola et al., 2020[10]). Other studies included participants with similar baseline characteristics (Grapow et al., 2015[4]; Plestis et al., 2018[14]; Ler et al., 2021[7]). The studies of Beute et al., Perin et al. and Grapow et al. did not provide information regarding strategies employed to reduce the effect of confounding factors (Beute et al., 2018[1]; Perin et al., 2019[13]; Grapow et al., 2015[4]). All studies did not provide follow up details (Supplementary information, Tables 1, 2). The RCT by Etiway et al. did not provide information about blinding of the treatment allocators and outcome assessors (Etiwy et al., 2018[3]).

### Statistical analysis

RevMan (version 5.4.1; Copenhagen: The Nordic Cochrane Centre, The Cochrane Collaboration) was used to conduct the meta-analysis. The outcomes of interest were provided as Risk Ratios (RR) with 95 % confidence intervals (CIs) and were aggregated using an inverse variance weighted random-effects model. Forest plots were used to graphically display the pooled analyses. MD and 95 % CIs were used to present the continuous outcomes of interest. Inverse variance weighted random-effects model was used to pool MD and 95 % CI. We used the median value where mean was unavailable. Difference in means between the baseline and post-intervention measurement was calculated when the change from baseline was not reported. The SD for change was derived from the baseline and the follow up, assuming their correlations were 0.5. The Higgins I^2^ was utilized to assess heterogeneity between trials. A 25-50 % number was regarded as low, 50-75 % moderate, and >75 % serious. In all cases, a P-value less than 0.05 was considered significant.

## Results

### Search results and baseline characteristics

**Figure 2:**
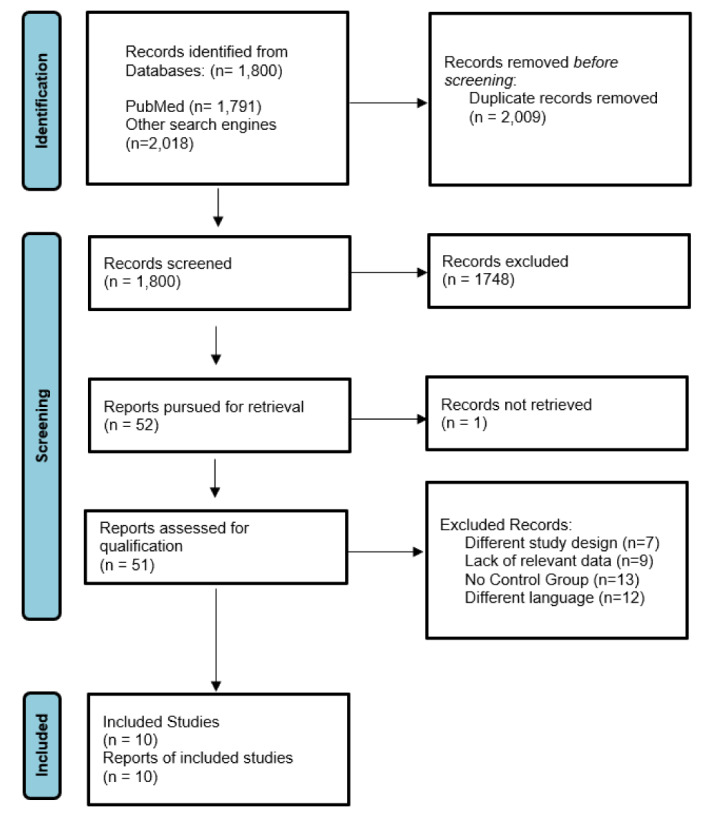
PRISMA 2020 flow diagram for new systematic reviews which included searches of databases and registers only (Page et al., 2021)

The PRISMA flow chart below summarizes the search and study selection process (Figure 2(Fig. 2); Reference in Figure 2: Page et al., 2021[12]). Initial search yielded a total of 1800 results. After screening and removal of duplicates, 51 articles were assessed for eligibility. Among those, seven of the studies had a different study design, nine studies did not report relevant outcomes of interest, thirteen of them did not have a control group, and twelve studies were not in English language. A total of 2 RCTs and 8 cohorts were included in the final analysis (Grapow et al., 2015[4]; Beute et al., 2018[1]; Etiwy et al., 2018[3]; Lee et al., 2018[6]; Loberman et al., 2018[8]; Plestis et al., 2018[14]; Sabik et al., 2018[16]; Perin et al., 2019[13]; Morgant et al., 2020[11]; Ler et al., 2021[7]).

A total of 1,411 participants were included in our study amongst which 721 were randomized to COR-KNOT while 690 participants were grouped into hand-tied knots. Table 1(Tab. 1) (References in Table 1: Beute et al., 2018[1]; Grapow et al., 2015[4]; Ler et al., 2021[7]; Loberman et al., 2018[8]; Morgant et al, 2020[11]; Perin et al., 2019[13]; Plestis et al., 2018[14]; Sabik et al., 2018[16]) and 2(Tab. 2) (References in Table 2: Etiwy et al., 2018[3]; Lee et al., 2018[6]; Ler et al., 2021[7]) summarize the baseline characteristics of included studies. Table 3(Tab. 3) contains the baseline characteristics of the study population of Ler et al. (2021[7]), as this particular study did not differentiate between minimally invasive cardiac surgery and open heart surgery. Table 4(Tab. 4) (References in Table 4: Beute et al, 2018[1]; Etiwy et al, 2018[3]; Grapow et al, 2015[4]; Lee et al, 2018[6]; Ler et al, 2021[7]; Loberman et al, 2018[8]; Perin et al, 2019[13]; Plestis et al, 2018[14]; Sabik et al, 2018[16]) summarizes the study details of the included studies, including the surgical procedure performed.

### Outcomes

#### Aortic cross-clamp Time (AXT)

**Figure 3:**
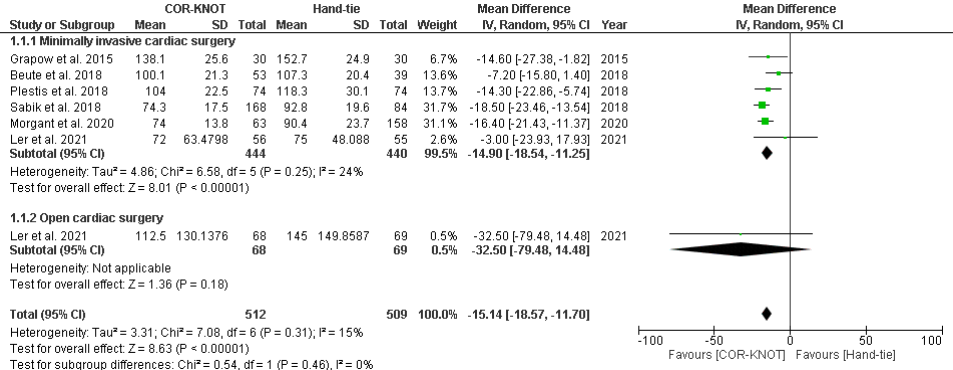
Forest plot comparing AXT in the COR-KNOT group vs Hand-tied

A total of six studies reported aortic cross-clamp time among patients with valvular disorder. We performed a subgroup analysis to compare the AXT, which included minimally invasive surgery and open cardiac surgery. Analysis revealed a significant difference in AXT between COR-KNOT versus hand-tied knots [MD -15.14, 95 % CI (-18.57, -11.70), P<0.00001, I²=15%] (Figure 3(Fig. 3); References in Figure 3: Beute et al., 2018[1]; Grapow et al., 2015[4]; Ler et al., 2021[7]; Morgant et al., 2020[11]; Plestis et al., 2018[14]; Sabik et al., 2018[16]).

#### Cardiopulmonary bypass (CPB) Time

**Figure 4:**
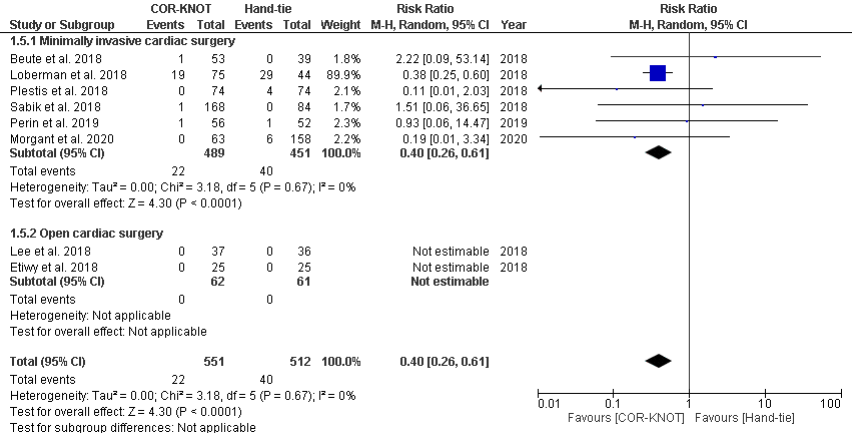
Forest plot comparing CPB time in the COR-KNOT group vs Hand-tied

Six studies reported CPB time as an outcome. A subgroup analysis was done to compare the CPB time, which included minimally invasive surgery and open cardiac surgery. Our meta-analysis showed a significant difference in CPB time between COR-KNOT versus hand-tied knots [MD -12.38, 95 % CI (-14.99, -9.77), P<0.00001, I²=0%] (Figure 4(Fig. 4); References in Figure 4: Beute et al., 2018[1]; Etiwy et al., 2018[3]; Lee et al., 2018[6]; Loberman et al., 2018[8]; Morgant et al., 2020[11]; Perin et al., 2019[13]; Plestis et al., 2018[14]; Sabik et al., 2018[16]).

#### Valvular regurgitation

**Figure 5:**
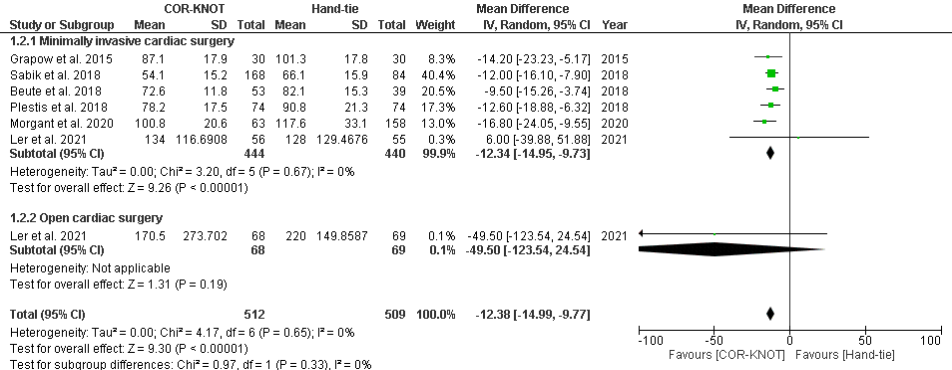
Figure 5: Forest plot comparing the incidence of valvular regurgitation in the COR-KNOT group vs Hand-tied

A total of eight studies reported valvular regurgitation as an outcome. A subgroup analysis was performed to compare the incidence of valvular regurgitation which included minimally invasive surgery and open cardiac surgery. Analysis yielded a significant difference in the incidence of valvular regurgitation between COR-KNOT versus hand-tied knots [RR 0.40, 95 % CI (0.26, 0.61), P<0.0001, I²=0%] (Figure 5(Fig. 5); References in Figure 5: Beute et al., 2018[1]; Grapow et al., 2015[4]; Ler et al., 2021[7]; Morgant et al., 2020[11]; Plestis et al., 2018[14]; Sabik et al., 2018[16]).

#### Mortality

**Figure 6:**
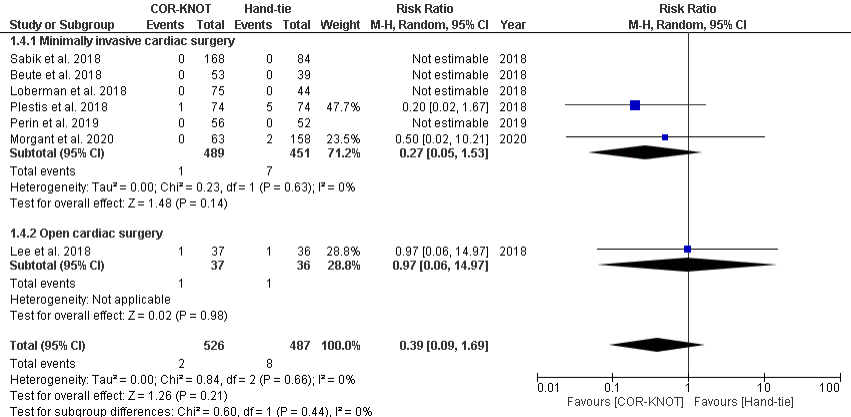
Forest plot comparing mortality in the COR-KNOT group vs Hand-tied

A total of seven studies reported mortality as an outcome. A subgroup analysis was performed to compare mortality which included minimally invasive surgery and open cardiac surgery. This meta-analysis reported no significant difference between the use of COR-KNOT versus hand-tied knot in preventing mortality [RR 0.39, 95 %CI (0.09, 1.69), P=0.44, I²=0%] (Figure 6(Fig. 6); References in Figure 6: Beute et al., 2018[1]; Lee et al., 2018[6]; Loberman et al., 2018[8]; Plestis et al., 2018[14]; Sabik et al., 2018[16]; Perin et al., 2019[13]; Morgant et al., 2020[11]).

#### Prolonged Ventilatory Support

**Figure 7:**
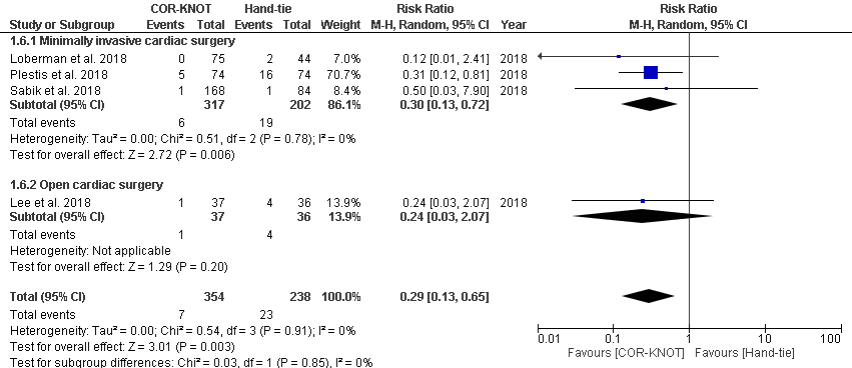
Figure 7: Forest plot comparing the need for prolonged ventilator support in the COR-KNOT group vs Hand-tied

Four studies with a total of 592 patients reported prolonged ventilatory support as an outcome of interest. Significantly lower rates of prolonged ventilator support were seen in patients sutured with COR-KNOT after valvular surgery when compared with those sutured with hand-tied knots [RR 0.29, 95 %CI (0.13, 0.65), P=0.003, I²=0%] (Figure 7(Fig. 7); References in Figure 7: Lee et al., 2018[6]; Loberman et al., 2018[8]; Plestis et al., 2018[14]; Sabik et al., 2018[16]).

#### Atrial Fibrillation

**Figure 8:**
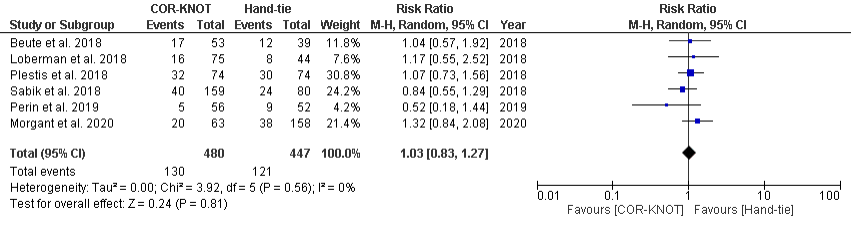
Forest plot comparing the incidence of atrial fibrillation in the COR-KNOT group vs Hand-tied

A total of five studies reported atrial fibrillation among patients undergoing valvular surgery. This meta-analysis reported no significant difference between the use of COR-KNOT versus hand-tied knot in preventing postoperative atrial fibrillation [RR 1.03, 95 %CI (0.83, 1.27), P=0.81, I²=0%] (Figure 8(Fig. 8); References in Figure 8: Beute et al., 2018[1]; Loberman et al., 2018[8]; Plestis et al., 2018[14]; Sabik et al., 2018[16]; Perin et al., 2019[13]; Morgant et al., 2020[11]).

#### Changes in Postoperative Left Ventricular Ejection Fraction (LVEF)

**Figure 9:**
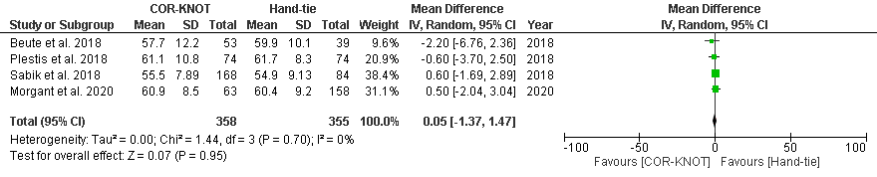
Forest plot comparing postoperative LVEF in the COR-KNOT group vs Hand-tied

Three studies with 492 patients reported no significant difference in postoperative LVEF between COR-KNOT versus hand-tied knots [MD 0.05, 95 % CI (−1.37, 1.47), P = 0.95, I^2^=0%] (Figure 9(Fig. 9); References in Figure 9: Beute et al., 2018[1]; Plestis et al., 2018[14]; Sabik et al., 2018[16]; Morgant et al., 2020[11]).

#### Renal failure

**Figure 10:**
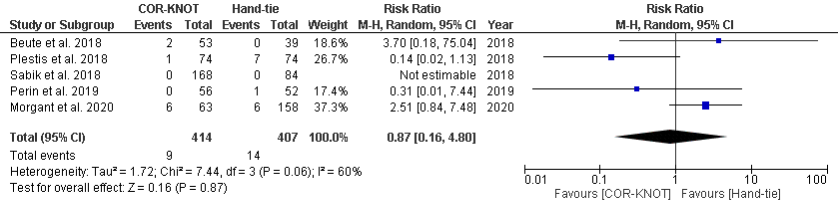
Forest plot comparing incidence of renal failure in the COR-KNOT group vs Hand-tied

A total of four studies comprising 600 patients reported renal failure as an outcome. No statistically significant difference was seen between COR-KNOT versus hand-tied knots in terms of incidence of renal failure [RR 0.87, 95% CI (0.16, 4.80), P = 0.87, I^2^=60%] (Figure 10(Fig. 10); References in Figure 10: Beute et al., 2018[1]; Plestis et al., 2018[14]; Sabik et al., 2018[16]; Perin et al., 2019[13]; Morgant et al., 2020[11]).

## Discussion

Valve repair and/or replacement continues to be one of the most common procedures for adults undergoing cardiac surgery. Two techniques are available to secure prosthetic valves and rings: traditional hand-tying and automated fasteners. Automated fasteners, exemplified by the COR-KNOT^@^ by LSI Solutions^®^, have emerged as an innovative approach to shorten prosthetic valve implantation time (Sazzad et al., 2021[19]) The present meta-analysis aimed at assessing the efficacy of COR-KNOT devices compared to hand-tied sutures in valve surgery.

Our findings demonstrated a significant reduction in both AXT and CPB times with the utilization of the COR-KNOT device, showcasing the potential for improved surgical efficiency. This reduction in operative time aligns with the benefits of automation (Loberman et al., 2018[8]; Salmasi et al., 2019[18]). Sazzad et al. also found a similar relationship between the use of COR-KNOT devices and reduction in AXT (MD = -14.36) and CPB time (MD = -11.74) (Sazzad et al., 2021[19]). Importantly, this advantage could lead to decreased exposure to the potential risks inherent in longer operations, potentially mitigating postoperative morbidity and mortality risks (Salmasi et al., 2019[18]; Sazzad et al., 2021[19]; Cody et al., 2021[2]).

Intriguingly, while the reduction in operative time was evident, our analysis did not reveal a significant difference in the incidence of atrial fibrillation between surgeries employing COR-KNOT devices and those using hand-tied knots. This suggests that the automated fasteners did not introduce an additional risk factor for postoperative atrial fibrillation, a common complication associated with cardiac surgeries. Therefore, practitioners can be reassured that the adoption of COR-KNOT devices does not appear to contribute to an increased risk of this particular complication. The previous meta-analysis by Sazzad et al. did not identify a correlation between postoperative atrial fibrillation and COR-KNOTs (Sazzad et al., 2021[19]).

Notably, the most significant advantage of using the COR-KNOT device was observed in the context of valvular regurgitation. Our analysis revealed a significant decrease in the incidence of valvular regurgitation when COR-KNOT device was used. This finding is also supported by Sazzad et al. (2021[19]) (RR = 0.40). It should also be noted, however, valve perforation followed by valvular regurgitation has been reported by two recent case reports following COR-KNOT devices use. Nevertheless, the failure was suspected to result from lack of experience with COR-KNOT deployment and can be prevented by being vigilant while operating with automated fastener and orienting and placing them away from native valve and or prosthetic leaflets (Salmasi et al., 2019[18]).

In terms of mortality, our study did not identify a significant difference between the two techniques. This finding suggests that the use of COR-KNOT devices is not associated with an increased mortality compared to hand-tying. However, it is important to note that the study did not identify a significant advantage in terms of mortality prevention either, indicating that further investigation is necessary to comprehensively assess the impact of automated fasteners on other relevant outcomes. Salmasi et al. also concluded that there was no significant difference in terms of 30-day mortality rate between conventional knot-tying and COR-KNOT, after analyzing several RCTs and retrospective studies (Salmasi et al., 2019[18]).

The reduction in the duration of ventilatory support among patients with COR-KNOT could be attributed to the reduced AXT and CPB time. Such benefits may contribute to shorter hospital stay and decrease other morbidities associated with prolonged intubation, which are essential factors in improving overall patient outcomes and resource utilization.

Our study's analysis of changes of postoperative LVEF did not reveal a significant difference between COR-KNOT and hand-tying. This suggests that both techniques maintain comparable cardiac function in the postoperative period. 

Lastly, the analysis of renal failure also did not yield a statistically significant difference between COR-KNOT and hand-tying. While the incidence of renal failure did not significantly vary between the two techniques, it is essential to recognize that this outcome can be influenced by multiple factors beyond the suturing method, including preoperative patient comorbidities and perioperative care.

Even though the study demonstrated compelling advantages, it is important to recognize the limitations and/or drawbacks that could be associated with both techniques. Thus, a comprehensive evaluation of the safety profile of both methods is needed prior to accepting the COR-KNOT on a wider scale.

### Limitations

The current study has the following limitations. The study focused on RCTs and cohorts, which may introduce variability and heterogeneity. The study was also limited by the small amount of literature available on postoperative outcomes of COR-KNOT device use. Future research is needed before accepting the COR-KNOT device on a wider scale. It is also important to note that the included studies had some degrees of bias that may affect the quality of evidence presented.

## Conclusion

In conclusion, this study contributes valuable insights into the ongoing discourse surrounding optimal techniques for knot securement during heart valve surgery. Our findings suggest that COR-KNOT device offers notable advantages in terms of reduced operative time and valvular regurgitation compared to hand-tying. However, we also recognize the need for caution in interpreting these results. The new studies have also not contributed to some of the outcomes (AXT, CPB time, valvular regurgitation, prolonged ventilator support, renal failure and postoperative LVEF) previously discussed in Sazzad et al's work, thus, there continue to be a need for further investigation and research in this field to derive a better conclusion for these outcomes and determine the potential for wide-spread use of automated fasteners in cardiac surgery. As the field continues to evolve, future research could delve into long-term outcomes, patient-specific factors, and the learning curve associated with transitioning to automated fasteners, ultimately refining our understanding of their place in cardiac valvular surgery.

## Declaration

### Conflict of interest

The corresponding author S.M.S is a consultant to Artivion, Abbott, and JOMDD.

### Ethics approval

Not applicable.

### Funding

No funding was available to the authors.

### Acknowledgments

None.

### Data availability statement

All relevant data are within the manuscript.

## Supplementary Material

Supplementary information

## Figures and Tables

**Table 1:**
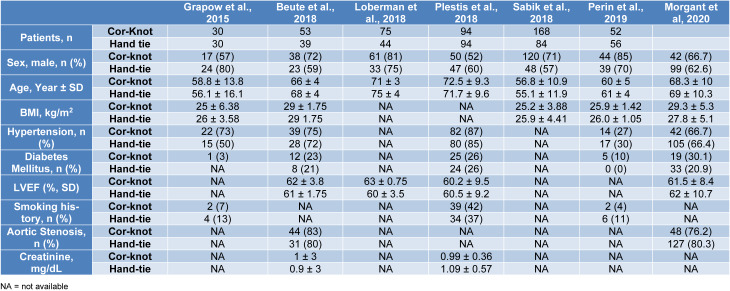
Baseline characteristics of the participants undergoing minimally invasive heart surgery (except Ler et al., 2021)

**Table 2:**
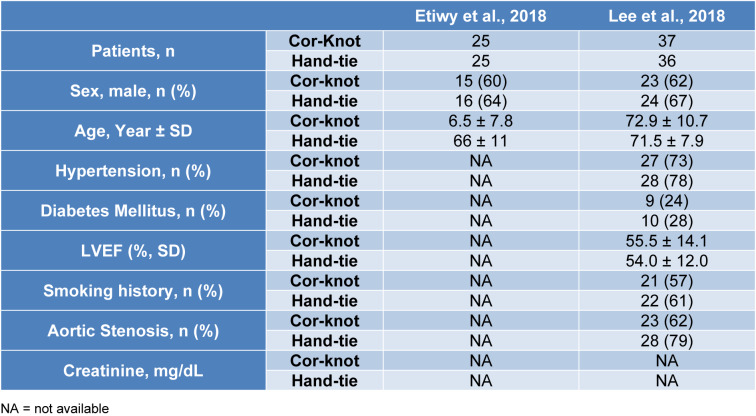
Baseline characteristics of the participants undergoing open heart surgery (except Ler et al., 2021)

**Table 3:**
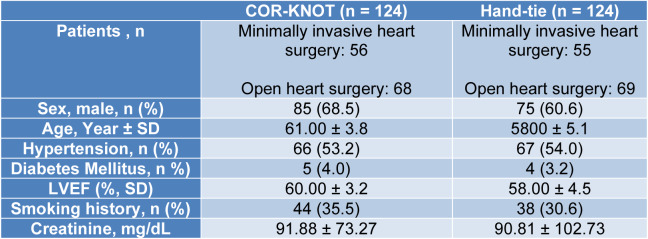
Baseline characteristics of the participants in Ler et al., 2021

**Table 4:**
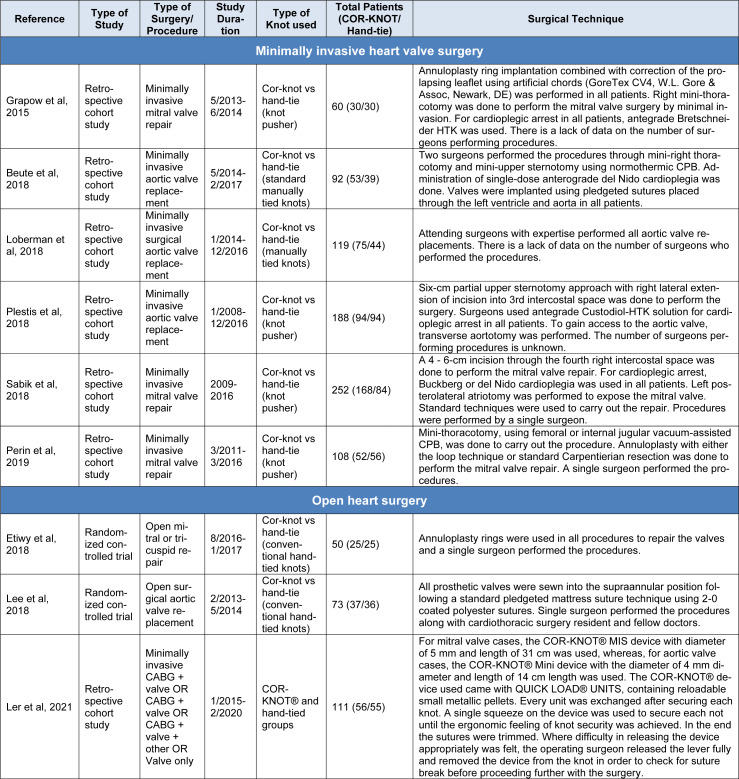
Characteristics of included studies

**Figure 1:**
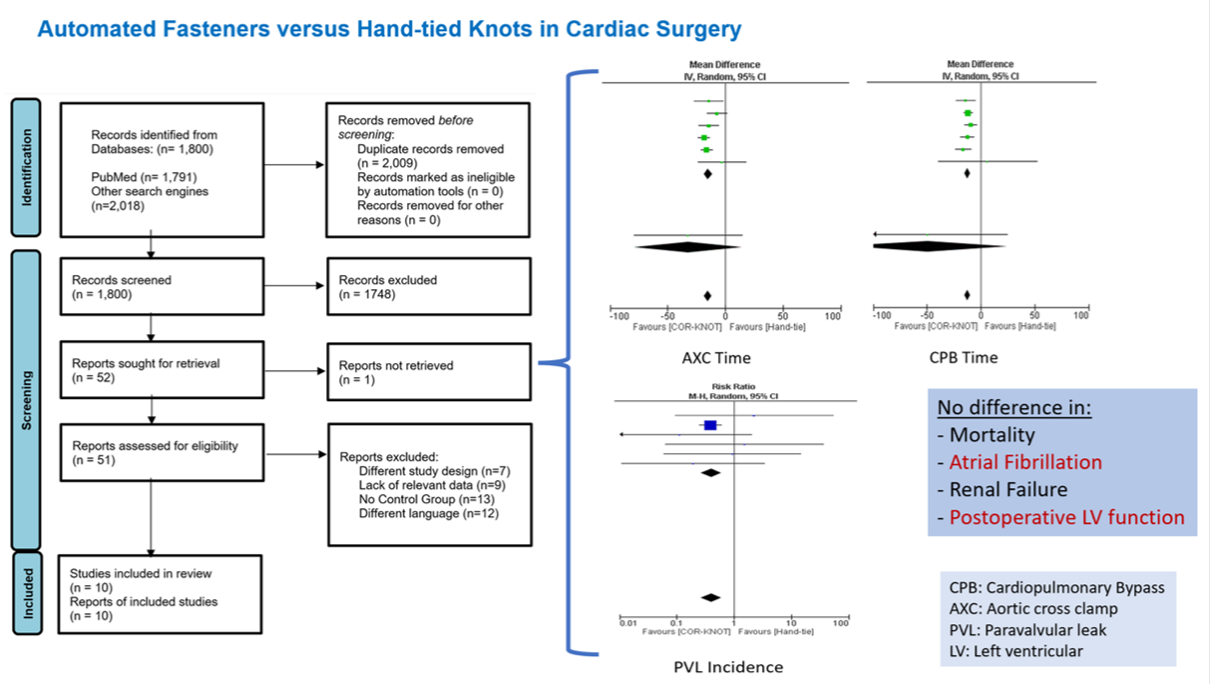
Graphical abstract
