# Supplementary material for: Efficacy of automated fasteners versus hand-tied knots in cardiac surgery: a systematic review and meta-analysis
Source: EXCLI J. 2024 Feb 6;23:198–211. doi: 10.17179/excli2023-6885 (PMC10938240; doi:10.17179/excli2023-6885)
Supplement: Supplementary information [file EXCLI-23-198-s-001.pdf]

EXCLI Journal 2024;23:198-211 – ISSN 1611-2156
Received: December 13, 2023, accepted: January 09, 2024, published: February 06, 2024

Supplementary information to:

Review article:

EFFICACY OF AUTOMATED FASTENERS VERSUS HAND-TIED KNOTS IN CARDIAC SURGERY: A SYSTEMATIC REVIEW AND META-ANALYSIS

Zoaib Habib Tharwani[a], Muhammad Abdul Qadeer[b], Ali Abdullah[b], Rubab Ali[b], Muhammad Ahmed Chaudhary[b], Shurjeel Uddin Qazi[a], Sameh M. Said[c,d*]

[a] Department of Medicine, Dow University of Health Sciences, Karachi, Pakistan
[b] Department of Medicine, Jinnah Sindh Medical University, Karachi, Pakistan
[c] Division of Pediatric and Adult Congenital Cardiac Surgery, Maria Fareri Children's Hospital, Westchester Medical Center, Valhalla, New York 10696, USA
[d] New York Medical College, 100 Woods Road, Valhalla, New York 10595, USA

* **Corresponding author:** Sameh M. Said, Professor of Surgery and Pediatrics, New York Medical College, Chief, Division of Pediatric and Adult Congenital Cardiac Surgery, Maria Fareri Children's Hospital, Department of Surgery, Westchester Medical Center, 100 Woods Road, Valhalla, New York, USA 10595; Department of Cardiothoracic Surgery, Faculty of Medicine, Alexandria University, Alexandria, Egypt.
Phone: 914-493-6696, Fax: 914-493-3853, E-mail: Sameh.Said@wmchealth.org

https://dx.doi.org/10.17179/excli2023-6885

This is an Open Access article distributed under the terms of the Creative Commons Attribution License (http://creativecommons.org/licenses/by/4.0/).

EXCLI Journal 2024;23:198-211 – ISSN 1611-2156
Received: December 13, 2023, accepted: January 09, 2024, published: February 06, 2024

Supplementary Table 1: Quality assessment of included studies using JBI critical appraisal checklist for cohort studies

Studies	Baseline characteristics	Similar exposure status	Exposure measured reliably	Identification of confounding factors	Strategies dealing with confounding factors	No outcome at the start	Measurement of outcome reliability	Follow up	Follow up complete or not	Strategies for incomplete follow up	Appropriate statistical methods
**Beute et al., 2018**	No	Yes	Yes	No	No	Yes	Yes	No	No	No	Yes
**Loberman et al., 2018**	Unclear	Yes	Yes	Yes	Yes	Yes	Yes	No	No	No	Yes
**Plestis et al., 2018**	Yes	Yes	Yes	Yes	Yes	Yes	Yes	No	No	No	Yes
**Sabik et al., 2018**	No	Yes	Yes	Yes	Yes	Yes	Yes	No	No	No	Yes
**Perin et al., 2019**	No	Yes	Yes	Yes	Unclear	Yes	Yes	No	No	No	Yes
**Grapow et al., 2015**	Yes	Yes	Yes	No	No	No	Unclear	No	No	No	Yes
**Ler et al., 2021**	Yes	Yes	Yes	Yes	Yes	Yes	Yes	No	No	No	Yes
**Morgant et al., 2020**	Yes	Yes	Yes	Yes	Yes	Yes	Yes	No	No	No	Yes

EXCLI Journal 2024;23:198-211 – ISSN 1611-2156
Received: December 13, 2023, accepted: January 09, 2024, published: February 06, 2024

Supplementary Table 2: Quality assessment of included studies using JBI critical appraisal checklist for RCTs

Studies	Randomization method reported?	Treatment group allocation	Baseline	Blinding of participants	Treatment Delivering Blinding	Outcome Assessers Blinding	Same Treatment	Follow up	Intention to treat	Outcome Measurement	Outcome Reliability	Statistical Analysis	Appropriate Trial Design
**Etiway et al., 2018**	Yes	No	Yes	Yes	No	No	Yes	No	Yes	Yes	Yes	Yes	Yes
**Lee et al., 2018**	No	No	No	Unclear	No	No	Unclear	No	No	No	Unclear	Yes	No
